# Supplementary material for: A Functional Model for Structure Learning and Parameter Estimation in Continuous Time Bayesian Network: An Application in Identifying Patterns of Multiple Chronic Conditions
Source: IEEE Access. Author manuscript; Available in PMC 2022 Apr 1. (PMC8975131; doi:10.1109/access.2021.3122912)
Supplement: supplemental [file NIHMS1755415-supplement-supplemental.pdf]

Appendices

A Model Training and Testing Procedure

Step by Step Training & Testing Procedure:

1. The number of patients considered for the analysis is 257,633.
2. Data Pre-processing: PCA is used to reduce the number of exogenous (risk factors) to one variable. This operation is only performed for the case of the functional CTBN model. For MTBN and LRMCL the exogenous variables are used directly without using any kind of transformation or reduction.
3. Model Implementation:
   (a) The functional CTBN model is implemented as discussed in the manuscript (based on Equation (12)). Likelihood score is used for optimization of the tuning parameter (λ) of the adaptive group regularization.
   (b) MTBN model is implemented as discussed in Faruqui et al. [4].
   (c) LRMCL model is implemented as discussed in Alaeddini et al. [19].
4. The model is trained using the training data. The data structures are formatted as per algorithms requirements.
5. The Area Under the Curve (AUC) of the Receiver Operating Characteristic (ROC) function based on 10-fold cross-validation is used to compare the performance of the competing algorithms.

B ICD-9 Codes

The ICD-9 codes for the disease conditions mentioned in the manuscript can be found in Table [A].

Table A: ICD-9 codes for the conditions used in the manuscript.

Condition	ICD-9 Codes
TBI	800, 801, 803, 804, 850, 851, 852, 853, 854, 905.0, 907.0, 950.1, 950.2, 950.3, 959.01, 959.9, 310.2, V15.52
PTSD	309.81
Back Pain	720, 721, 722, 724, 737, 738, 739, 756, 805, 839, 847
Substance Abuse	291.xx, 292.xx, 303.x, 304.x, 305.0, 305.2, 305.3, 305.4, 305.5, 305.6, 305.7, 305.8, 305.9
Depression	296.2x, 296.3x, 311
